# Portal vein aneurysm associated with arterioportal fistula after hepatic anterior segmentectomy: Thought-provoking complication after hepatectomy

**DOI:** 10.1186/s40792-018-0465-9

**Published:** 2018-06-15

**Authors:** Yusuke Kimura, Tomohide Hori, Takafumi Machimoto, Tatsuo Ito, Toshiyuki Hata, Yoshio Kadokawa, Shigeru Kato, Daiki Yasukawa, Yuki Aisu, Yuichi Takamatsu, Taku Kitano, Tsunehiro Yoshimura

**Affiliations:** Department of Digestive Surgery, Tenri Hospital, 200 Mishima-cho, Tenri City, Nara Prefecture 632-8552 Japan

**Keywords:** Shunt, Arterioportal fistula, Portal vein aneurysm, Complication, Hepatectomy, Intervention radiology

## Abstract

**Background:**

Few cases of postoperative arterioportal fistula (APF) have been documented. APF after hepatectomy is a very rare surgery-related complication.

**Case presentation:**

A 62-year-old man was diagnosed with hepatocellular carcinoma in segments 5 and 8, respectively. Anterior segmentectomy was performed as a curative surgery. Each branch of the hepatic artery, portal vein, and biliary duct for the anterior segment was ligated together as the Glissonean bundle. The patient was discharged on postoperative day 14. Three months later, dynamic magnetic resonance imaging showed an arterioportal fistula and portal vein aneurysm. Surprisingly, the patient did not have subtle symptoms. Although a perfect angiographic evaluation could not be ensured, we performed angiography with subsequent interventional radiology to avoid sudden rupture. Arteriography was immediately performed to create a portogram via the APF from the stump of the anterior hepatic artery, and portography clearly revealed hepatofugal portal vein flow. Portography also showed that the stump of the anterior portal vein had developed a 40-mm-diameter portal vein aneurysm. Selective embolization of the anterior hepatic artery was accomplished in the whole length of the stump of the anterior hepatic artery, and abnormal blood flow through the APF was drastically reduced. The portal vein aneurysm disappeared, and portal flow was normalized. Dynamic computed tomography after embolization clearly demonstrated perfect interruption of the APF. The patient maintained good health thereafter.

**Conclusions:**

Post-hepatectomy APFs are very rare, and some appear to be cryptogenic. Our thought-provoking case may help to provide a possible explanation of the causes of post-hepatectomy APF.

## Short description

Arterioportal fistula (APF) is a rare condition, and some APFs appear to be cryptogenic. Few cases of postoperative APF have been documented, and APF after hepatectomy is a very rare surgery-related complication. We herein presented a thought-provoking case of surgery-related APF after hepatectomy. Our case may be informative with respect to explaining the possible causes of APF after hepatectomy.

## Background

An arterioportal fistula (APF) or shunt is a rare condition. An APF causes portal hypertension [1] and sometimes results in life-threatening events (e.g., liver failure, hepatic encephalopathy, and variceal bleeding) [2–4] requiring surgical treatment, interventional radiology (IVR), and endovascular therapy [2, 5]. The known etiologies of APF include trauma, iatrogenic causes (e.g., biliary drainage, percutaneous biopsy, and radiofrequency ablation), congenital disease, malignant tumors, and splanchnic artery aneurysm rupture [1–4, 6–8].

Major hepatectomy is currently a feasible and safe therapeutic option for liver disease [9, 10], although some postoperative complications are intractable (e.g., biliary leakage, portal thrombosis, and refractory ascites) [11–13]. Fatal complications have been reported, such as liver failure, secondary portal hypertension, and abnormal hemostasis [13, 14], and massive bleeding from varices or aneurysms will affect the postoperative course after hepatectomy [13, 15].

APF is a very rare complication after hepatectomy [16], although cases of APF after gastrectomy and laparoscopic cholecystectomy have been documented [17, 18]. To the best of our knowledge, only one case of APF after hepatectomy has been reported [16]. We herein report a thought-provoking case of APF after anterior segmentectomy. We also discuss a possible explanation for the cause of APF in this case.

## Case presentation

**Fig. 1:**
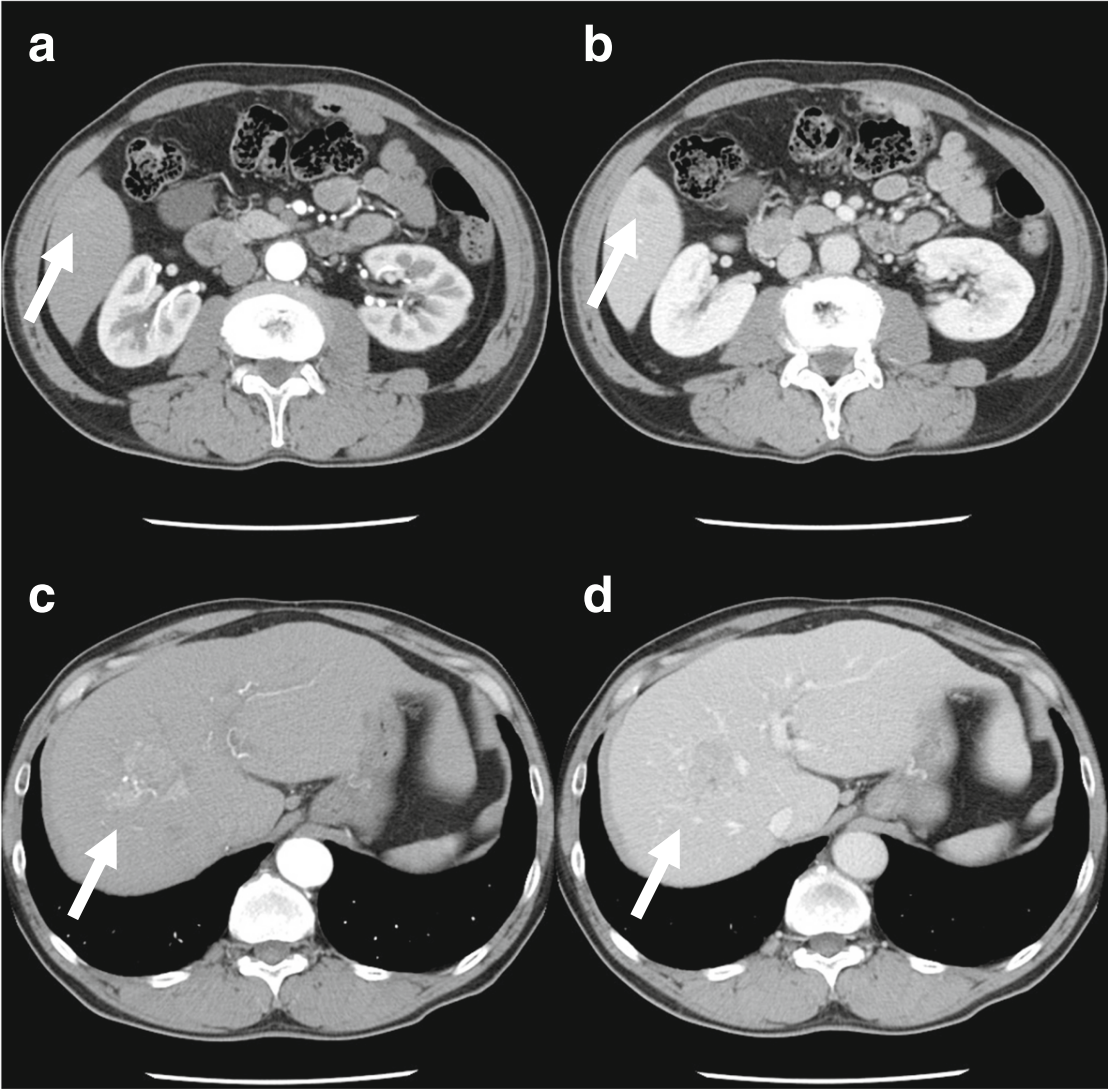
Dynamic CT findings before surgery. Dynamic CT revealed two tumors (arrows) located in **a**, **b** segment 8 and **c**, **d** segment 5. **a**, **c** Findings in the early phase. **b**, **d** Findings in the delayed phase

**Fig. 2:**
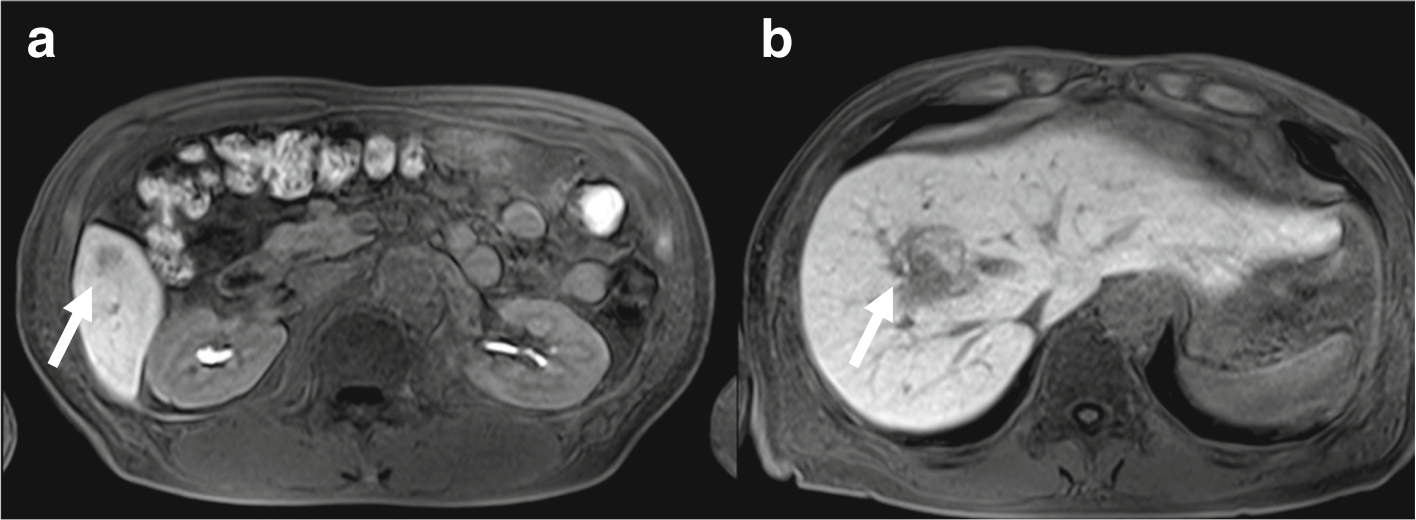
RI findings enhanced by Gadolinium-ethoxybenzyl-diethylenetriamine pentaacetic acid before surgery. Enhanced MRI revealed two tumors (arrows) located in **a** segment 8 and **b** segment 5

**Fig. 3:**
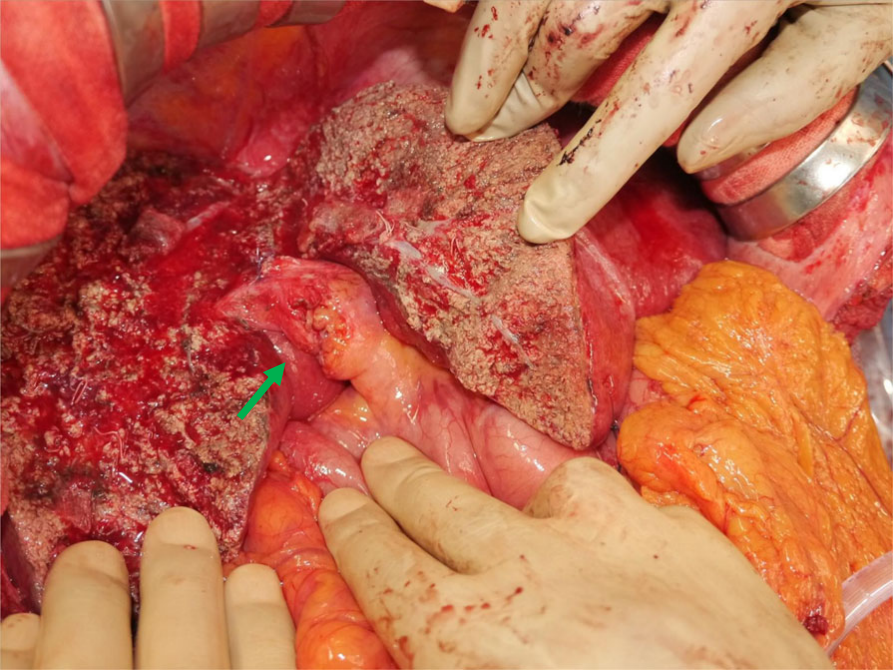
Intraoperative findings of FSTG. Anterior segmentectomy was performed. Each branch of the hepatic artery, portal vein, and biliary duct were ligated together as the Glissonean bundle (so-called FSTG) (arrow)

A 62-year-old man with chronic hepatitis C was referred by his physician to our hospital for surgical treatment of hepatocellular carcinoma. Imaging findings on enhanced computed tomography (CT) and dynamic magnetic resonance imaging (MRI) revealed two tumors located in segments 5 and 8, respectively (Figs. 1 and 2). Although the alpha-fetoprotein level was within the reference range, the serum level of prothrombin induced by the absence of vitamin K or antagonist-II was high (530 mAU/ml). After a preoperative evaluation based on a three-dimensional (3D) imaging study, anterior segmentectomy was performed. Each branch of the hepatic artery, portal vein, and biliary duct for the anterior segment were ligated together as the Glissonean bundle (so-called, fully simultaneous transection of the Glissonean pedicle [FSTG]) (Fig. 3). Perihilar FSTG involved a transfixation suture by using an absorbable thread. The tumor in segment 8 was in contact with the middle hepatic vein. However, this tumor was well-encapsulated, and the tumor and vein were easily dissectable. The patient’s postoperative course was uneventful, and he was discharged on postoperative day 14.

**Fig. 4:**
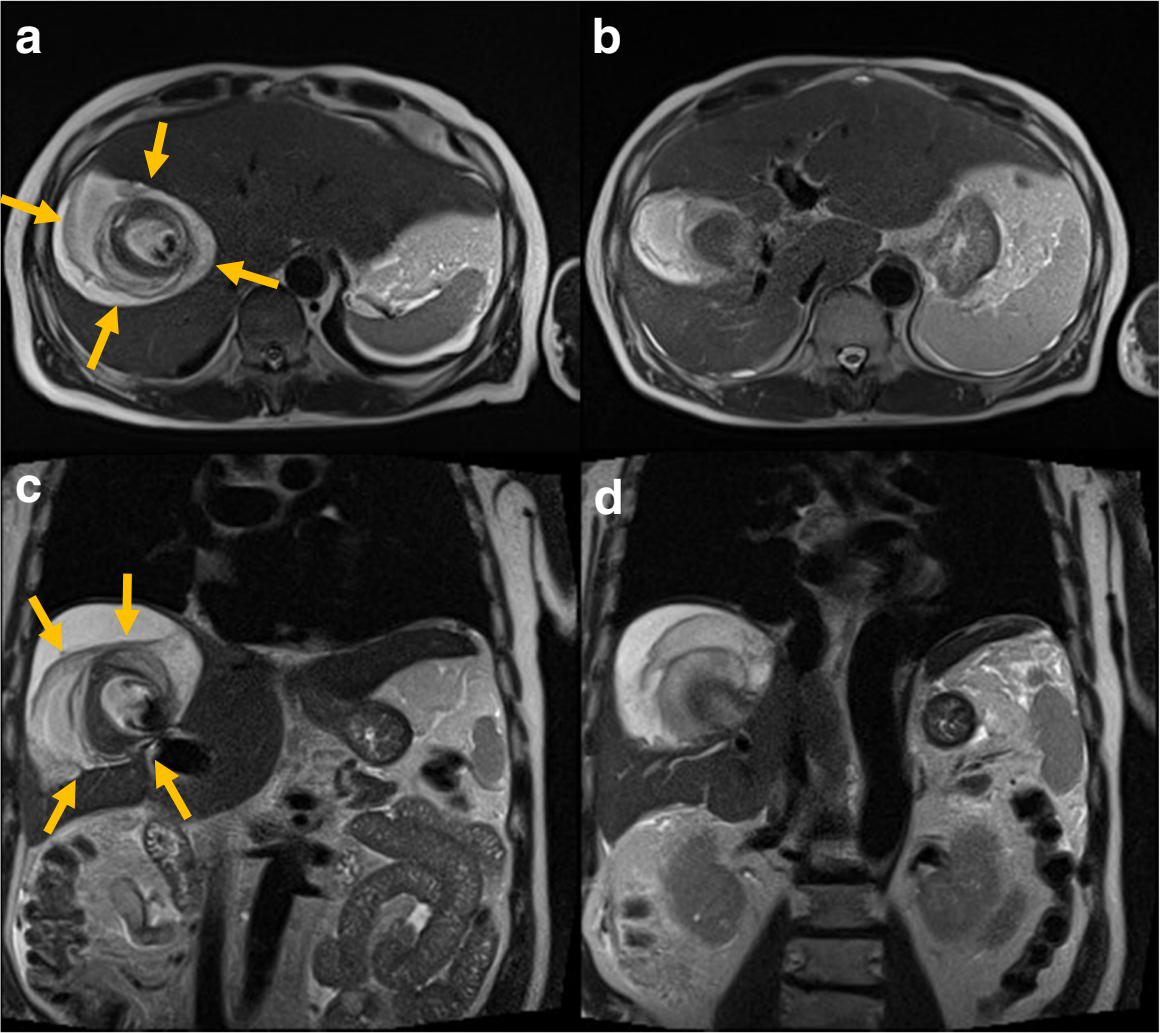
Dynamic MRI findings 3 months after surgery. **a**, **b** Axial images of dynamic MRI. **c**, **d** Coronal images of dynamic MRI. An arterioportal fistula and portal vein aneurysm were incidentally detected. Layers of old and subacute hematomas were clearly observed, and these layers surrounded the aneurysm **(**arrows**)**. We suspected a pseudoaneurysm based on these MRI findings

Three months later, dynamic MRI was performed to check for intrahepatic recurrence, and no imaging findings of recurrence were observed. However, an arterioportal fistula and portal vein aneurysm were incidentally detected (Fig. 4). Layers of old and subacute hematomas were clearly observed, and these layers surrounded the aneurysm. Surprisingly, the patient did not have subtle symptoms and showed no episodes of pain, ascites, liver dysfunction, or other abnormalities. We suspected a pseudoaneurysm at that time. Although a perfect angiographic evaluation could not be ensured, IVR was needed to avoid sudden rupture and possible death. Therefore, we decided to attempt IVR after evaluation of the vessels on dynamic CT, and transcatheter arterial embolization was proposed thereafter.

**Fig. 5:**
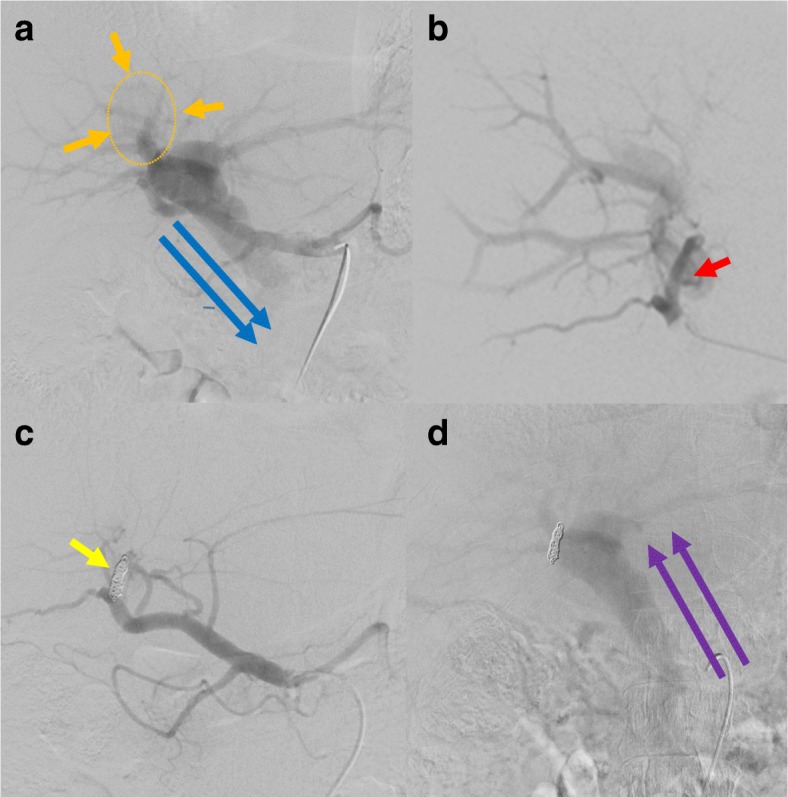
Findings of angiography. **a** Angiography via the celiac artery was performed to create a portogram via the APF, and portography clearly revealed hepatofugal flow of the portal vein **(**blue arrows**)**. Portography also showed that the stump of the anterior portal vein had developed a PVA with a diameter of 40 mm **(**orange arrows**)**. **b** Selective catheterization of the common hepatic artery clearly demonstrated the APF at the stump of the anterior branches (red arrows). A definitive diagnosis of PVA due to APF was made. An adequate length of APF to perform embolic therapy was confirmed. **c** Selective embolization of the anterior hepatic artery was accomplished by placement of several titanium coils (yellow arrow). Blood flow through the APF was drastically reduced. **d** Arteriography via the superior mesenteric artery showed hepatopetal portal flow (purple arrows)

First, angiography via the celiac artery was performed. Arteriography was subsequently used to create a portogram via this APF, and portography clearly revealed hepatofugal flow of the portal vein. Portography also showed that the stump of the anterior portal vein had developed a portal vein aneurysm (PVA) with a diameter of 40 mm (Fig. 5a). Selective catheterization of the common hepatic artery was then performed. This arteriography clearly demonstrated a fistula between the hepatic artery and portal vein (i.e., APF) at the stump of the anterior branches (Fig. 5b). Based on these angiography findings, we definitively diagnosed PVA due to an APF, not a pseudoaneurysm.

Next, an adequate length of APF to perform embolic therapy was confirmed to avoid any occlusion and disturbance at the bifurcation of the right hepatic artery (Fig. 5b). Selective embolization of the anterior hepatic artery was then accomplished by placing several titanium coils in the whole length of the stump of the anterior hepatic artery. Finally, the flow of blood through the APF was drastically reduced (Fig. 5c). Arteriography via the superior mesenteric artery showed a remarkable restoration of portal venous flow, and hepatopetal portal flow was clearly observed (Fig. 5d).

**Fig. 6:**
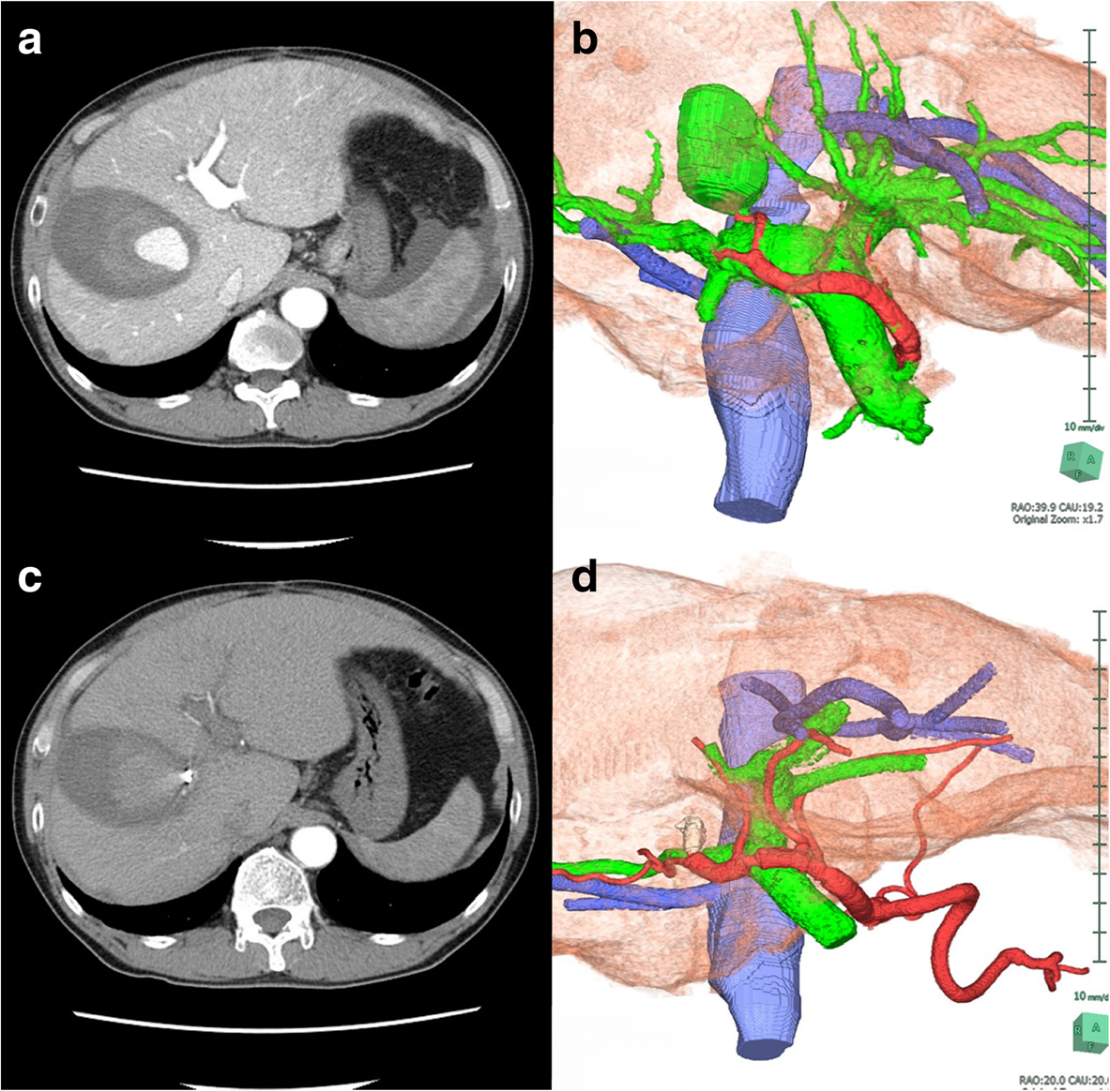
Imaging findings before and after IVR. **a**, **b** Imaging findings before IVR. Prior to IVR, the APF and PVA were detected by **a** dynamic CT and **b** 3D images. **c**, **d** Imaging findings after IVR. Three days after embolization, **c** dynamic CT and **d** 3D images clearly demonstrated both perfect interruption of the APF and disappearance of the PVA

Complete closure of the APF could be estimated by additional expansion of the metallic coils over time after IVR. Dynamic CT and 3D images 3 days after embolization clearly demonstrated perfect interruption of the APF and disappearance of the PVA (Fig. 6).

Imaging studies and serum biomarkers showed no evidence of recurrence. At the time of this study, the patient was good in health and had been reintegrated into society.

## Conclusions

Causes of APF include trauma, iatrogenic causes (e.g., biliary drainage, percutaneous biopsy, and radiofrequency ablation), congenital disease, malignant tumors, and splanchnic artery aneurysm rupture [1–4, 6–8]. Some APFs appear to be cryptogenic. Surgery-related APFs are rare, although cases of APF after gastrectomy and laparoscopic cholecystectomy have been reported [17, 18]. To the best of our knowledge, however, only one case of APF after hepatectomy has been documented, and this previous case occurred in a 3-month-old infant after right trisegmentectomy [16]. The infant had been successfully treated by super-selective embolization using titanium coils. His artery, portal vein, and bile duct were ligated, respectively. This approach was distinct from our approach, FSTG.

A simple question arose in the present case: Is FSTG dangerous during hepatectomy? FSTG is currently a safe and reproducible hepatectomy technique because of its simplicity, and this technique has therefore become a standard method during major hepatectomy [9, 10]. In our institution, we also employ perihilar FSTG during major hepatectomy for hepatocellular carcinoma, metastatic tumors, and benign diseases. In the present case, we understood that it is difficult to discuss the mechanism of APF development. We considered that our case was an agnogenic APF and that a relation between perihilar FSTG and resultant APF was also unclear. Our procedures of perihilar FSTG involved a transfixation suture, and we here speculated that perihilar FSTG might have been a possible cause of the APF. We also speculated that other possible causes (e.g., technical error) were observed, and recognized that a responsible cause of our APF was still obscure. We had not experienced similar cases of APF after hepatectomy accompanied by FSTG, although we found no reports of FSTG-related APF.

Although our patient was asymptomatic, CT detected a small amount of ascites. APFs, especially those on the proximal side (e.g., intrahepatic or perihilar Glissonean pedicle), often result in refractory symptoms of portal hypertension (such as gastrointestinal bleeding, ascites, and diarrhea) [6]. In the present case, a definitive diagnosis of APF was made only 3 months after hepatectomy, and we considered that the reason why the patient had no symptoms was the prompt diagnosis of APF followed by adequate IVR. If prompt diagnosis followed by adequate therapy had failed, his portal hypertension would likely produce intractable symptoms over time. A simple question arose in the present case: Why symptoms of portal hypertension did not appear in this case? We speculated one possible reason was that APF was developed not at the main trunk level but at the stump of anterior Glissonean pedicle which was located at relatively peripheral lesion compared with main trunk.

Therapeutic strategies for APFs include surgery (e.g., partial hepatectomy and ligation of the related hepatic artery) and IVR (e.g., transarterial embolization). Our patient had an adequate length between the stump of the anterior hepatic artery and the bifurcation of the right hepatic artery, and it was considered suitable for transarterial embolization. We suggest that angiography should be considered as the first-choice imaging technique to elucidate the details of APFs and subsequently determine the optimal therapy [19].

Glissonean pedicle transection (i.e., FSTG) is now routinely employed during hepatectomy worldwide. We consider this maneuver to be very useful during major hepatectomy. The occurrence of APF after surgery is considered to be low [16–18]. However, once an APF has developed, the patient will experience a poor clinical course accompanied by severe portal hypertension, and this intractable complication requires surgical or interventional treatment.

APF after hepatectomy is very rare, and some APFs appear to be cryptogenic. Our thought-provoking case may be informative in terms of providing a possible explanation of the causes of APF after hepatectomy.

## Abbreviations

3D: Three-dimensional
APF: Arterioportal fistula
CT: Computed tomography
FSTG: Fully simultaneous transection of the Glissonean pedicle
IVR: Interventional radiology
MRI: Magnetic resonance imaging
PVA: Portal vein aneurysm
